# Functional Differences in Muscle Architecture Across the Pelvis and Hind Limb of Primates

**DOI:** 10.1002/ajpa.70329

**Published:** 2026-07-31

**Authors:** Emma Guimaraes, Evie Vereecke, Ashleigh L. Wiseman

**Affiliations:** ^1^ Department of Anthropology University College London London UK; ^2^ Department of Development and Regeneration KU Leuven Campus Kulak Kortrijk Belgium; ^3^ McDonald Institute for Archaeological Research University of Cambridge Cambridge UK

**Keywords:** dissection, locomotion, muscle architecture, musculoskeletal, primates

## Abstract

**Objectives:**

Understanding the architectural diversity of primate musculature is essential for interpreting locomotor function and reconstructing evolutionary adaptations. This study examines whether interspecific differences in pelvic and hind limb muscle architecture reflect functional differentiation among major muscle groups.

**Materials and Methods:**

We present detailed dissection data from six primate species, alongside a large sample of additional specimens collected from previous studies. We quantify fascicle length, pennation angle, muscle mass, and physiological cross‐sectional area across the hind limb and pelvis in each species. We assessed whether differences in muscle architecture were consistent across functional groups and taxa. Differences in muscle architecture were analyzed across functional groups, muscles, and species, with architectural specialization interpreted in functional terms, e.g., reflecting relative emphasis on force, power, displacement, or generalist/stabilizing roles.

**Results:**

Our results reveal conserved configurations in load‐bearing muscles, but marked divergence in others, particularly in knee and hip musculature. Notably, muscle specialization was more conserved between species with similarity in their ecology/locomotor mode than between those who are closely related, indicating that muscle architecture is closely linked to habitual hind limb function.

**Discussion:**

These findings suggest that patterns of pelvic and hind limb muscle specialization across primates are driven primarily by ecological and locomotor function, rather than evolutionary relatedness. The dataset provides a detailed, anatomically grounded resource for future comparative and biomechanical studies of primate hind limb function.

## 1 Introduction

Understanding muscle architecture is key to interpreting how animals generate and control movement. Primates are especially interesting in this regard, as they employ a diverse range of locomotor modes, from suspensory climbing and brachiation to terrestrial quadrupedalism. This diversity in movement is reflected in the architecture of their limb muscles, which provides a framework for investigating functional specialization and the mechanical basis of locomotor behavior. In this study, we focus primarily on hominoid primates, with macaques included as a cercopithecoid outgroup to provide comparative context. Although all hominoids, except for modern humans, are capable of generalized arboreality (Crompton et al. 2010, 2008), differences in locomotor ecology (Aerts et al. 2018; Channon et al. 2009; Granatosky 2018; King et al. 2026; Myatt et al. 2012; Thorpe and Crompton 2005) suggest that architectural specializations in muscles may vary between predominantly terrestrial taxa, such as chimpanzees, bonobos and gorillas (Hunt et al. 1996), and those that are predominantly arboreal, such as orangutans and gibbons (Thorpe and Crompton 2006; Vereecke et al. 2005). Based on these ecological distinctions, one might expect more terrestrial species to exhibit muscle architectures that are optimized for force production and postural support, whilst arboreal species favor configurations that enhance velocity and range of motion (also referred to as displacement). Such patterns have been documented across mammals, where terrestrial taxa have muscles with relatively greater force‐generating capacity, and arboreal taxa exhibit architectural features associated with increased excursion and mobility (Böhmer et al. 2018; Leischner et al. 2018; Taverne et al. 2018). Despite these general patterns, quantitative data comparing muscle architecture across different hominoid species remain limited, particularly for hind limb muscles, making it difficult to link architecture to locomotor specialization.

The primate musculoskeletal system exhibits a conserved structural plan (Diogo et al. 2010; Diogo et al. 2019; Diogo et al. 2017; Molnar and Diogo 2021); yet, within this framework there is considerable variation in muscle dimensions, fiber lengths, physical cross‐sectional area (PCSA), and pennation angles across taxa (Payne et al. 2006a). It is the composition of these elements within a muscle that directly determines a muscle's capacity for force production, contraction velocity, and range of motion (Granatosky and Ross 2020; Nagano et al. 2005; Rome et al. 1988; Seth et al. 2018, 2011). Long, parallel‐fibred muscles typically favor rapid or large‐range movements, while short, pennate muscle with a large PCSA is optimized for force production (Charles et al. 2022; Zajac 1989). Such variation reflects the tuning of muscle function to meet differing biomechanical demands, depending on their functional role (Thorpe et al. 1999).

Muscle pennation plays a key role in determining a muscle's force‐generating capacity (Zajac 1989). Pennate muscles pack more fibers into a given volume, increasing a muscle's PCSA and thus maximal force, but this comes at the cost of reduced fiber length and excursion (Eng et al. 2018; Gans 1982). In contrast, parallel‐fibred muscles, with fibers running largely along the muscle's line of action, favor longer fibers and greater shortening velocity, supporting rapid or large‐range movements. Across hominoids, most hind limb muscles exhibit low pennation angles, typically < 30° (Ledoux et al. 2001; Payne et al. 2006a; Thorpe et al. 1999; Vereecke et al. 2005). Historically, variation in pennation has had a relatively modest effect on PCSA estimates for comparative studies with limited species (Lieber 2022; Martin et al. 2020; Rockenfeller et al. 2024). Because PCSA is calculated as muscle mass divided by fiber length, scaled by the cosine of the pennation angle (Zajac 1989), angles below ~20° result in cos(θ) values close to 1 and therefore have only a minor influence on PCSA estimates, whereas larger pennation angles substantially increase force‐generating capacity. New research with a larger comparative sample quantifying pennation alongside fascicle length and muscle volume can therefore provide unexplored insight into the functional specialization of individual muscles and the mechanical strategies underlying locomotor diversity.

Comparative analyses of muscle architecture have revealed that certain muscle groups are subject to stronger functional and evolutionary influences than others (Polk 2002). For example, distal limb muscles often exhibit greater specialization linked to substrate contact and propulsion (Payne et al. 2006a), and this higher specialization is associated with greater interspecific variability, whereas proximal limb muscles are largely similar across taxa and are more conserved (Charles et al. 2016; Deane et al. 2024, 2020; Leischner et al. 2018; Myatt et al. 2012). A similar proximal–distal pattern is also observed in limb segment proportions, with proximal elements tending to be more constant and distal elements more variable (Molnar et al. 2017). Although several studies have quantified hind limb muscle architecture in hominoids (Aerts et al. 2018; Marchi et al. 2018; Payne et al. 2006a, 2006b; Vereecke et al. 2005), broad comparative datasets spanning multiple lineages remain limited, and few have explicitly tested how variation in muscle form translates into differences in mechanical function.

Here, we build upon these previous comparative efforts by examining the relationship between muscle architecture and functional specialization, testing whether architectural parameters, such as fiber length, pennation angle, and PCSA, correspond to adaptations for force‐ or velocity‐based performance. Understanding these links is critical because it allows us to move beyond descriptive anatomy to evaluate the mechanical roles that muscles play in shaping locomotor capabilities. By identifying whether specific muscle groups are tuned for generating high forces or for facilitating rapid limb excursions, we can better understand how muscle design supports the diverse locomotor strategies observed across hominoid species.

In this study, we test three predictions derived from functional interpretations of muscle architecture. First, if pennation angle materially affects estimates of force‐generating capacity, then incorporating pennation should significantly alter PCSA estimates relative to calculations assuming parallel fibers. Second, if muscle architecture reflects locomotor specialization, then species occupying different ecological niches should exhibit systematic differences in architectural traits (fiber length, PCSA, and pennation angle). Third, if functional specialization is reflected in architectural design, then muscles should show associations between fiber length and PCSA consistent with force‐, velocity‐, or displacement‐biased roles across taxa.

## 2 Materials and Methods

### 2.1 Extant Data Collection

The right leg and pelvis of six fresh‐frozen primate species were dissected at the Jan Palfijn Anatomy Lab, KU Leuven, Belgium (Table 1). All specimens were opportunistically collected post‐mortem from European zoos, with no animals sacrificed for the study. Ethical approval was provided by the Animal Ethics Committee of the KU Leuven, Belgium (approval codes M005/2023, M006/2023), and all procedures complied with EU regulations. All specimens were frozen shortly after death and stored at −20°C. The specimens were thawed at room temperature for 1–3 days prior to the dissections. Some specimens underwent several freeze–thaw cycles due to data collection for multiple research projects (i.e., Van Beesel et al. 2025).

**TABLE 1:** Details of specimens included in the study.

Family	Species	*n*	Specimen ID	Source	Sex	Age	Information available	Analyses performed
*Cercopithecidae*	*Macaca mulatta*	1	127 [KU Leuven, Belgium]	This study	M	Adult	Full pelvis and limb dissected; all parameters measured	Comparisons of pennation, tendon parameters, and functional differences in architecture
*Hylobatidae*	*Symphalangus syndactylus*	1	22NL302953/20 [Diergaarde Amersfoort]	This study	M	26.2		
	*Hylobates lar*	3	FR2101100017‐K [Sigean, France]	This study	M	44.7		
			Mo	Vereecke et al. (2005)	M	25	PCSA and fiber lengths only	Comparisons of functional differences in architecture
			Haf	Payne et al. (2006b)	F	16		
*Hominidae*	*Pongo abelii*	2	Oaf	Myatt et al. (2011)	F	45		
			129: EAZA/2953 [Planckendael, Belgium]	This study	F	23	Full pelvis and limb dissected; all parameters measured	Comparisons of pennation, tendon parameters, and functional differences in architecture
	*Gorilla g. gorilla*	5	131: 23NL320497/20 [Blijdorp, Rotterdam]	This study	M	27.1		
			Gam	Myatt et al. (2011)	M	30	PCSA and fiber lengths only	Comparisons of functional differences in architecture
			Gsm	Myatt et al. (2011)	M	18		
			Oishi_gorilla	Oishi et al. (2009)	F	40		
			Gm	Payne et al. (2006b)	M	33		
	*Gorilla g. graueri*	1	Gj	Payne et al. (2006b)	M	30		
	*Pan paniscus*	4	Ppam	Myatt et al. (2011)	M	22		
			Pp	Payne et al. (2006b)	M	29.6		
			De	Vereecke et al. (2005)	M	29		
			Dz	Vereecke et al. (2005)	F	31		
	*Pan troglodytes*	2	130: 22NL302953/20 [Zoo Antwerp, Belgium]	This study	F	40.5	Lower limb dissected; pelvis unavailable	Comparisons of pennation, tendon parameters, and functional differences in architecture
			Ptsm	Myatt et al. (2011)	M	11	PCSA and fiber lengths only	Comparisons of functional differences in architecture
	*Homo sapiens*	10	Subject01	Charles et al. (2019)	M	23		
			Subject02	Charles et al. (2019)	M	26		
			Subject03	Charles et al. (2019)	M	29		
			Subject04	Charles et al. (2019)	F	26		
			Subject05	Charles et al. (2019)	F	23		
			Subject06	Charles et al. (2019)	F	35		
			Subject07	Charles et al. (2019)	F	25		
			Subject08	Charles et al. (2019)	F	26		
			Subject09	Charles et al. (2019)	M	26		
			Subject10	Charles et al. (2019)	M	34		

*Note:* *N* represents the total number of specimens per species.

Additional data were obtained from published sources (Charles et al. 2019; Myatt et al. 2011; Oishi et al. 2009; Payne et al. 2006b) (Table 1). In total, our sample includes 30 specimens, spanning nine species, primarily in the *Homininidae* family with some from the *Hylobatidae* (lesser apes) and a cercopithecid ( *Macaca mulatta* ), which is included as an outgroup in this study. This sample is also previously described in Wiseman et al. (2026).

Body mass could not be recorded for all dissected specimens because the upper limb and torso had been dissected for previous studies (Van Beesel et al. 2025). Only *Gorilla gorilla* (gorilla) had a known body mass at death (180 kg); the remaining masses were estimated (see below). Dissections focused on the right hind limb, except in *Hylobates lar* (gibbon) and *Pan troglodytes* (chimpanzee), for which the left hind limb was also examined. The gibbon right hind limb was affected by a pre‐mortem pathology, necessitating left hind limb dissection to obtain representative measurements. In the chimpanzee specimen, hip musculature was unavailable due to prior dissection, reducing the available musculature for this specimen. Variation in the number of muscles measured across specimens reflected genuine anatomical differences as detailed.

Muscles were identified by two observers using standard anatomical atlases and photographed in situ before removal. Origin and insertion homology was further determined according to species‐specific anatomical atlases (Diogo et al. 2013; Diogo et al. 2010, 2017; Diogo et al. 2019) and is extensively described in Supporting Information S2, as well as previously described in Wiseman et al. (2026). A single observer then measured and weighed each muscle, and architectural parameters, such as muscle and tendon length, muscle mass, fascicle length (FL), and pennation angle (θ), were recorded. FL was measured with calipers to an accuracy of ±0.01 mm, and muscle masses with precision scales to ±0.01 g. θ was measured using protractors as the angle between the fascicles and the line of action of the tendon. For each muscle, multiple FL and θ measurements (≈10 per muscle) were taken at different locations along the muscle, and the average was calculated to account for intramuscular variation (Charles et al. 2022; Cuff et al. 2023; Martin et al. 2020). These data were used to calculate muscle volume and PCSA. Muscle and tendon mass were initially measured together as a single dissected unit and then were physically separated and re‐measured as two units (tendon and belly). In total, we measured 48 muscles per hind limb (Table 2).

**TABLE 2:** Pelvis and hind limb muscles included in this study.

Muscle name	Abbrev.	Muscle name (cont.)	Abbrev. (cont.)
*M. adductor magnus* ^a^	AM^a^	*M. biceps femoris* (*long head*)	BFL
*M. adductor brevis*	AB	*M. biceps femoris* (*short head*)	BFS
*M. adductor longus*	AL	*M. semimembranosus*	SM
*M. adductor minimus*	AMin	*M. semitendinosus*	ST
*M. pectineus*	PECT	*M. popliteus*	POP
*M. gracilis*	GRA	*M. lateral gastrocnemius*	LG
*M. obturator externus*	ObtExt	*M. medial gastrocnemius*	MG
*M. obturator internus*	ObtInt	*M. vastus intermedius*	VI
*M. gluteus maximus* ^b^	GMax^b^	*M. vastus lateralis*	VL
*M. gluteus medius*	GMed	*M. vastus medialis*	VM
*M. gluteus minimus*	GMin	*M. tibialis anterior*	TA
*M. gemellus superior*	GemSup	*M. tibialis posterior*	TP
*M. gemellus inferior*	GemInf	*M. extensor hallucis longus*	EHL
*M. quadratus femoris*	QF	*M. extensor hallucis brevis*	EHB
*M. tensor fasciae latae*	TFL	*M. flexor hallucis longus* ^c^	FHL^c^
*M. piriformis*	PIRI	*M. flexor hallucis brevis*	FHB
*M. iliacus*	ILI	*M. flexor digitorum longus* ^c^	FDL^c^
*M. rectus femoris*	RF	*M. flexor digitorum brevis*	FDB
*M. sartorius*	SAR	*M. extensor digitorum longus*	EDL
*M. psoas major*	PMaj	*M. extensor digitorum brevis*	EDB
*M. psoas minor*	P_Min_	*M. adductor hallucis*	AH
*M. abductor hallucis*	AbH	*M. plantaris*	PLANT
*M. soleus*	SOL	*M. peroneus/fibularis brevis*	PB
*M. peroneus/fibularis longus*	PL	*M. abductor digiti minimi*	AbDm

^a^ Note that the AM had two bellies in some species (see: Section 3): the AMinf (inferior head) and AMsup (superior head).

^b^ The macaque gluteus superficialis was treated as homologous to the human gluteus maximus.

^c^ The m. flexor fibularis and m. flexor tibialis in some of the non‐human primates were mapped to the human FHL (digit 1) and FDL (digits 2–5) based on anatomy/function (see: Section 3).

Not all muscles and measurements were available across all species due to musculoskeletal differences, methodological constraints or were not made available in published literature (i.e., reports of tendons lengths for the human dataset were unavailable). Where data were not available, species or muscles are excluded from analysis (Table 2), and only available data are reported.

### 2.2 Body Mass Calculations for Missing Data

With the exception of the gorilla, for which body mass at death was recorded, body mass for the remaining dissected specimens was estimated using one of two approaches:

1. Convex hull estimation. For specimens with full‐body CT scans, body mass was estimated from the convex hull of segmented body regions following established protocols (Brassey and Sellers 2014; Coatham et al. 2021). This method was applied to the *Macaca mulatta* (macaque), *Symphalangus syndactylus* (siamang), gibbon, and *Pongo abelii* (orangutan) specimens.
2. Long‐bone scaling. For the chimpanzee specimen, only a partial CT scan was conducted. Body mass was estimated using femoral and humeral measurements according to established allometric scaling relationships (Burgess et al. 2018).

### 2.3 Calculation of Muscle Parameters

Normalized muscle mass (M_norm_) and FL (*L*_*f*_) were calculated as follows (Alexander et al. 1981):

(1) $$M_{\mathrm{norm}}=m_{\text{muscle}}/\text{Body mass}$$

(2) $$L_{f}=\mathrm{FL}/{\text{Body mass}}^{1/3}$$

M_norm_ was subsequently compared between species. Next, each muscle's PCSA was calculated as (Zajac 1989):

(3) $$\text{PCSA}=\frac{\cos\left(\theta \right)\cdot m_{\text{muscle}}}{\rho \cdot L_{f}}$$

Normalized PCSA (PCSA_norm_) was subsequently calculated as follows:

(4) $${\text{PCSA}}_{\mathrm{norm}}=\text{PCSA}/{\text{Body mass}}^{2/3}$$

where *ρ* is tissue density of 1.060 kg m^−3^ (Mendez and Keys 1960), and $m_{\text{muscle}}$ is the muscle belly mass. Muscles were then grouped according to their main function (Charles et al. 2019, 2020; Herrel et al. 2025) (Table 3). Each muscle was assigned to a single functional category according to its primary action, ensuring that functional group masses summed to total hindlimb muscle mass and that categories were mutually exclusive. Body mass–normalized variables were used to enable interspecific comparison of relative muscle architectural traits across taxa differing in overall body size. While regression‐ or allometry‐based approaches can be used to explicitly model scaling relationships, these require substantially larger and more evenly distributed samples than are available in the present comparative dataset.

**TABLE 3:** Muscle functional groups for each joint in the primate hind limb, and constituent muscles.

Joint	Functional group	Muscles
Hip	Hip extensors	GMax, BFL, SM, ST
	Hip flexors	ILI, SAR, PMaj, P_Min_
	Hip abductors	GMed, GMin, TFL, PIRI
	Hip adductors	AM^a^, AB^b^, AL, PECT, Amin, GRA
	Hip rotators	GemSup, GemInf, ObtInt, ObtExt, PIRI, QF
Knee	Knee extensors	VI, VL, VM, RF
	Knee flexors	POP, BFS, BFL, GRA, PLANT
Ankle	Ankle dorsiflexors	TA, EHL, EDL
	Ankle plantarflexors	SOL, MG, LG, TP, PB, PL
Toe	MTP flexors	AbDm^c^, AbH^c^, FDB^c^, FDL, FHL
	MTP extensors	EHL, EHB, EDB

*Note:* Each muscle appears only once within a functional group, as each specimen provides a single set of architectural measurements (i.e., FL and PCSA) per muscle. Muscles are grouped according to their primary function, but for visualization purposes some distal muscles (e.g., EDL, FDL) may appear within broader functional categories rather than strict muscle antagonistic pairs.

^a^ AM had two bellies in the chimpanzee and the gorilla.

^b^ AB had two bellies in the gibbon. For visualization, both AM and AB bellies are plotted as separate data points but are labeled as AM or AB (*n* = 2 points per individual for these muscles).

^c^ AbDm and AbH were measurable only in the chimpanzee, gorilla, gibbon, and bonobo specimens, and FDB only in the gorilla and orangutan, due to dissection constraints in the remaining taxa. They were retained in functional space analyses but excluded from muscle mass and pennation statistics to maintain a consistent muscle set across species. For visualization purposes only, AbDm and AbH are displayed within the MTP flexor category.

### 2.4 Assessing Muscle Function From Architectural Parameters

Muscles were grouped according to primary function (Table 3).

Next, we used the ratio of $L_{f}$ to PCSA_norm_ to investigate the differences in muscle specializations between species. By doing so, muscles can be categorized according to their functional emphasis: force‐oriented (short fibers with large PCSA; plots in top left quadrant), displacement‐oriented (long fibers with small PCSA; plots in bottom right quadrant), or power‐oriented (intermediate to long fibers with moderate to high PCSA; plots in top right quadrant). Although it is not possible to define strict quantitative thresholds separating different regions of architectural space, broad functional patterns can be interpreted from the overall distribution of muscles within the $L_{f}$:PCSA_norm_ morphospace (e.g., Charles et al. 2022; De Ridder et al. 2025). Minor shifts in position within this space are expected due to methodological variation and biological noise in architectural measurements across specimens and estimation approaches. However, larger shifts in relative position reflect meaningful differences in inferred muscle function. In particular, substantial separation along the $L_{f}$ or PCSA_norm_ axes indicates a change in the balance between force‐ and displacement‐related specialization, with intermediate positions representing more generalized or power‐oriented architectural configurations. To allow direct comparison across data sources where pennation data were not available, pennation angle was omitted (assumed to be 0) when calculating PCSA_norm_ in all specimens. Functional space analyses combine muscle architectural data from our dataset and other published sources (Table 1). Importantly, these datasets differ in their treatment of pennation angle in PCSA estimation, with our dataset incorporating pennation angle and some sources (Payne et al. 2006a; Myatt et al. 2011) reporting PCSA under a parallel‐fibered assumption. All analyses therefore reflect broad comparative patterns in muscle architectural organization.

Tendon architecture was quantified using tendon length ratio. Tendon length ratio was defined as tendon length divided by total muscle–tendon unit (MTU) length. This ratio was included to characterize the relative contribution of tendinous versus contractile tissue along the limb and to capture proximal–distal variation in elastic versus contractile investment.

### 2.5 Statistical Analysis

Species differences in grouped mean pennation angles were tested using a one‐way ANOVA with species as a fixed effect. Post hoc pairwise comparisons were conducted using Tukey's HSD test to adjust for multiple comparisons. Analyses were performed in R (version 2025.09.2).

To evaluate the influence of pennation angle on PCSA, we compared values calculated assuming parallel fibers (i.e., no pennation correction) with those incorporating measured pennation angles. Accordingly, two PCSA values were calculated for each muscle: PCSA according to Equation (3), and parallel PCSA, assuming θ = 0. To assess the impact of including pennation on PCSA estimates, paired *t*‐tests were performed for each species dissected in this study (*N* = 6).

Tendon length ratios were analyzed using two‐way ANOVAs with species and joint as fixed effects, followed by Tukey's HSD tests for post hoc comparisons. All analyses were conducted in R (version 2025.09.2).

## 3 Results

### 3.1 Muscle Homology

Muscle attachment sites were broadly conserved across taxa, although several differences in presence and morphology were observed (Supporting Information S2; also see Wiseman et al. 2026), particularly at the hip and within the foot. Homology was determined through anatomical position and inferred function, supplemented by published descriptions when direct observation was ambiguous (Casteleyn et al. 2023; Channon et al. 2009; Diogo et al. 2019; Diogo et al. 2010; Diogo et al. 2019; Diogo et al. 2017; Molnar and Diogo 2021; Payne et al. 2006a, 2006b; Vereecke et al. 2005; Ziermann et al. 2021). In cases where exact homology was uncertain or questionable (e.g., GMax in hominoids and *M. gluteus superficialis* in macaques), homology was assumed based on anatomical and functional similarity (Diogo et al. 2019; Diogo et al. 2010; Diogo et al. 2019; Diogo et al. 2017). In non‐human primates, the digital flexors include *m. flexor fibularis* and *m. flexor tibialis*, which arise from the fibula and tibia, respectively, and insert via multiple tendons onto digits 1–5. These muscles are functionally equivalent to the human FHL (digit 1) and FDL (digits 2–5), and are referred to as such here, assuming direct homology based on their shared path and action (digital flexion and ankle plantarflexion).

Deep hip musculature showed the greatest variability: for instance, the *Mm. obturator* (ObtInt, ObtExt) and *gemelli* (GemSup, GemInf) complex differed in composition among species, with ObtInt occasionally absent or exhibiting additional bellies, and GemSup absent in some great apes (Supporting Information S2). The QF was sometimes fused with the deep portion of AM, suggesting a continuum in their functional role as lateral rotators.

Variation was also pronounced within the adductor compartment: the AB ranged from having a single to multiple bellies, and an additional AMin was present in some species (e.g., gorilla, chimpanzee) but missing in others, indicating possible lineage‐specific differentiation. The PECT was variably expressed or fused with AL, consistent with previous observations of its phylogenetic lability (Sigmon 1974).

In contrast, the superficial gluteal complex was relatively conserved in topology but differed in size and origin extent. The GMed exhibited broad variation in the dimensions of the tendon footprint at its attachment at the femoral head (greater trochanter). In macaques, the *m. gluteus superficialis* (homologous to the GMax of hominoids) was relatively small, whereas humans exhibited a marked posterior expansion in muscle belly. Overall, patterns of similarity in the deep and superficial hip musculature broadly reflected phylogenetic relatedness, with closer affinities between congeneric taxa (chimpanzees and gorillas) and greater divergence from the more distantly related (humans and macaques) (Supporting Information S2).

*M. scansorius* and *M. ischiofemoralis* were present only in select taxa, namely, chimpanzees, orangutans, gibbons, and siamangs, consistent with their known phylogenetic variability within Hominoidea. These muscles were not included in the assessments.

In general, muscle attachments to the greater and lesser trochanters and trochanteric fossa were identical in all species.

The muscles crossing the knee and ankle were generally conserved in overall morphology and attachment sites, although PLANT was variably absent in several species, in keeping with its known evolutionary reduction (Diogo et al. 2010, 2012, 2017, 2019; Diogo et al. 2013). In contrast, the foot and ankle musculature exhibited substantial variability, particularly among the digital flexors and extensors. The FHL and FDL showed variable patterns of fusion and tendon insertion, consistent with differing functional demands for pedal grasping in arboreal taxa versus propulsive force generation in more terrestrial forms (Supporting Information S2). Similarly, FDB and FHB displayed considerable morphological diversity and occasional fusion, underscoring the high degree of developmental plasticity in intrinsic foot muscles. The FDB was comparatively consistent in its proximal attachment, arising from the calcaneus in all examined taxa. However, distal morphology was less uniform: in chimpanzees and bonobos, its tendons showed partial fusion with FDL and/or FHL. Although FHL most commonly inserted onto the distal phalanx of the hallux, additional slips to digits II–IV were frequently observed in the great apes. Moreover, fusion between FHL and FDL occurred in chimpanzees, bonobos, siamangs, and gibbons. In some taxa, the FHL and FDL remained distinct, whereas in others they were partially or extensively fused, with insertion patterns varying across digits. For example, in gorillas, macaques, orangutans, and humans, the FDL inserted onto digits II–V. By comparison, both gibbons and siamangs lacked a tendon to digit V. Chimpanzees and bonobos exhibited clear fascial integration between FHL and FDL, indicating a degree of functional and/or developmental coupling between these muscles.

In the gibbon and siamang, the muscle bellies of the *Mm. peronei* were fused. However, there were two separate tendons inserting homologously with the other taxa.

**FIGURE 1:**
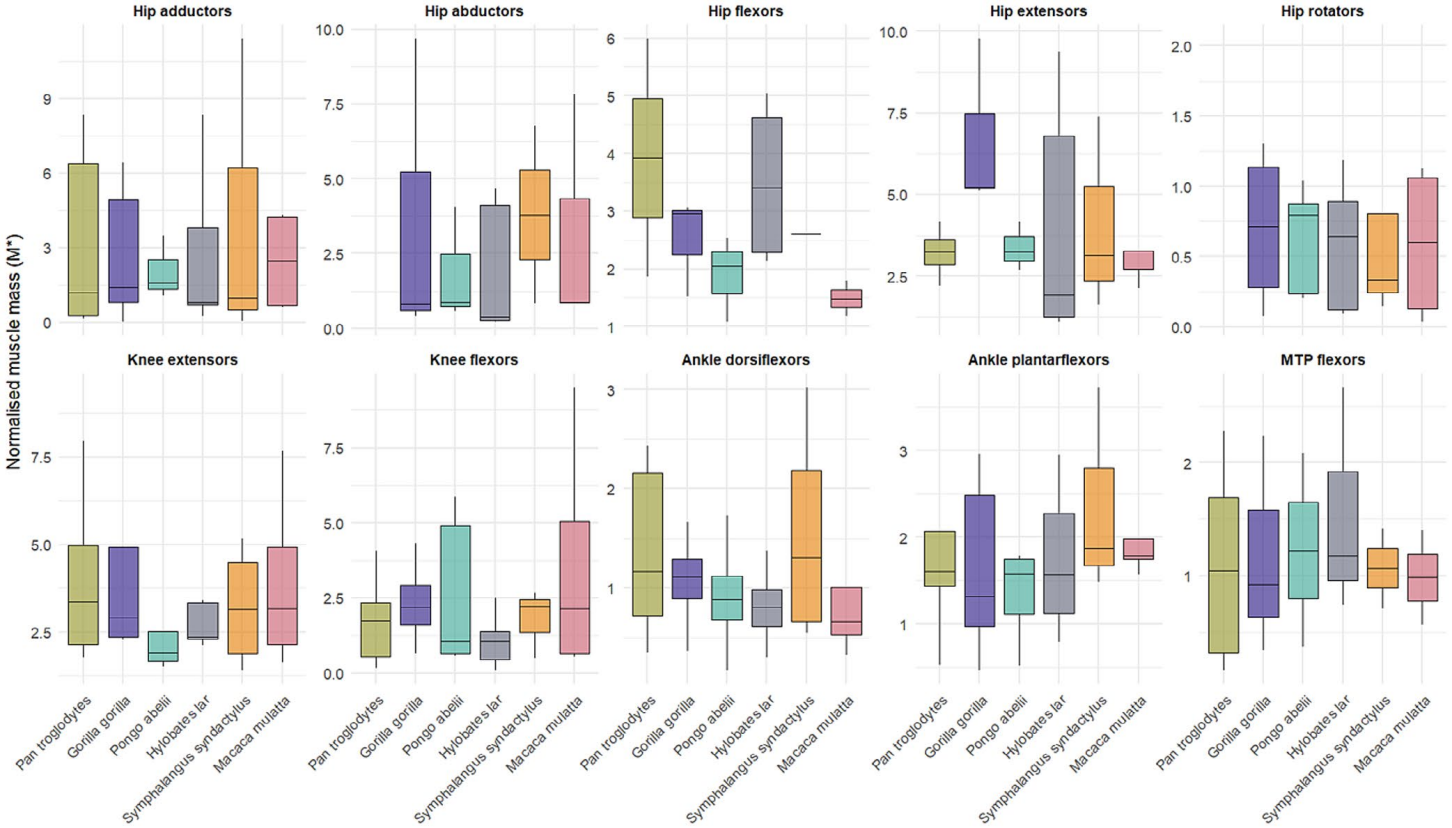
Normalized muscle mass of each muscle group. Only specimens dissected in this study are included (*n* = 6) due to availability of data.

Regarding the distribution of muscle mass, M_norm_ values were broadly comparable across species for the hip adductors, abductors, rotators, knee extensors and flexors, and for all ankle and foot musculature (Figure 1). Differences between species were generally small. Notable exceptions included an intraspecific variation of ankle dorsiflexor muscle mass in chimpanzees and siamangs, and in the mass of knee flexors in orangutans and macaques.

In contrast, the hip flexors and extensors showed clear interspecific divergence. Hip flexor normalized mass was substantially larger in chimpanzees and gibbons, and lowest in macaques. For the hip extensors, gorillas exhibited the largest normalized mass, while both gibbons and siamangs displayed wide ranges spanning relatively small to relatively large values.

### 3.2 Differences in Muscle Architecture

**FIGURE 2:**
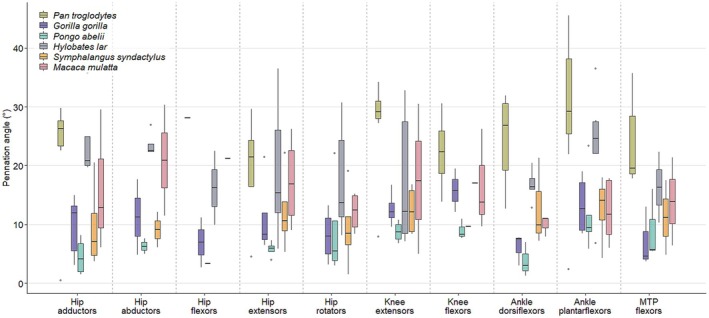
Boxplot comparing pennation angle across the primate hind limb in a sample of 19 homologous muscles (see: Supporting Information S1). Only specimens dissected in this study are included (*n* = 6). Data for chimpanzee pelvis is not available due to missing soft tissue during dissection. MTP extensors are grouped here with the ankle dorsiflexors due to small sample sizes.

Across the sampled primates (*n* = 6), pennation angle varied significantly among species when all muscles were considered together (one‐way ANOVA: *n* = 217 observations, df = 5, 211, *F* = 30.63, *p* < 2 × 10^−16^; Figure 2). Chimpanzees showed the highest overall pennation angles (mean 25.0° ± 9.33, *n* = 46) and were significantly more pennate than every other species (Tukey HSD: all *p* ≤ 0.01; e.g., chimpanzee–gorilla: Δ = 14.69°, 95% CI = [9.93, 19.45], *p* < 0.0001). When grouped by primary function, significant among‐species differences were observed, particularly in distal limb muscles. Species effects were detected in hip adductors (*F* = 4.58, *p* = 0.0051), hip abductors (*F* = 4.48, *p* = 0.034), knee extensors (*F* = 3.21, *p* = 0.024), ankle dorsiflexors (*F* = 8.62, *p* = 0.0003), ankle plantarflexors (*F* = 5.33, *p* = 0.0013), and MTP flexors (*F* = 3.32, *p* = 0.038), whereas hip flexors (*F* = 2.05, *p* = 0.353), hip extensors (*F* = 1.77, *p* = 0.164), hip rotators (*F* = 1.31, *p* = 0.308), and knee flexors (*F* = 1.69, *p* = 0.242) showed no statistically detectable species differences. In line with the overall pattern, chimpanzees tended to show the highest mean pennation in ankle plantarflexors (29.4° ± 12.1°; *n* = 10) and ankle dorsiflexors (24.5° ± 7.89°; *n* = 6), while orangutans remained lowest across most groups, including ankle dorsiflexors (3.77° ± 2.93°; *n* = 3) and knee extensors (8.78° ± 1.81°; *n* = 4).

Next, we included (assuming parallel fibers) and excluded pennation (assuming pennated fibers) in our PCSA estimates (Table 4). Including pennation angle in PCSA calculations significantly reduced PCSA estimates in all species except the orangutan, indicating that fiber orientation exerts a measurable effect on muscle architecture. Orangutans and chimpanzees showed no significant difference between parallel and pennate PCSAs estimates. Although the absolute differences in PCSA values were small, the consistent and taxon‐dependent pattern indicates that pennation angle exerts a systematic influence on architectural estimates rather than introducing random variation.

**TABLE 4:** *T*‐tests comparing PCSA with and without pennation angle.

Species	*N*	Parallel PCSA Mdn (IQR)	Full PCSA Mdn (IQR)	95% CI	*p*
Gorilla	37	6.11 (3.90)	6.09 (3.90)	8.26e‐07 to 2.40e‐06	**< 0.001**
Orangutan	33	3.70 (2.70)	3.67 (2.70)	−8.91e‐07 to 5.11e‐06	0.16
Siamang	28	5.75 (5.20)	5.55 (5.10)	7.13e‐07 to 3.22e‐06	**0.003**
Gibbon	27	6.84 (8.80)	6.28 (7.80)	4.49e‐06 to 1.94e‐05	**0.003**
Chimpanzee	25	5.19 (8.30)	4.73 (7.80)	2.03e‐06 to 1.86e‐05	0.017
Macaque	31	4.78 (7.70)	4.57 (7.70)	2.08e‐06 to 8.22e‐06	**0.002**

*Note:* *p*‐values in bold are statistically significant.

### 3.3 Differences in Muscle Function/Specialization

**FIGURE 3:**
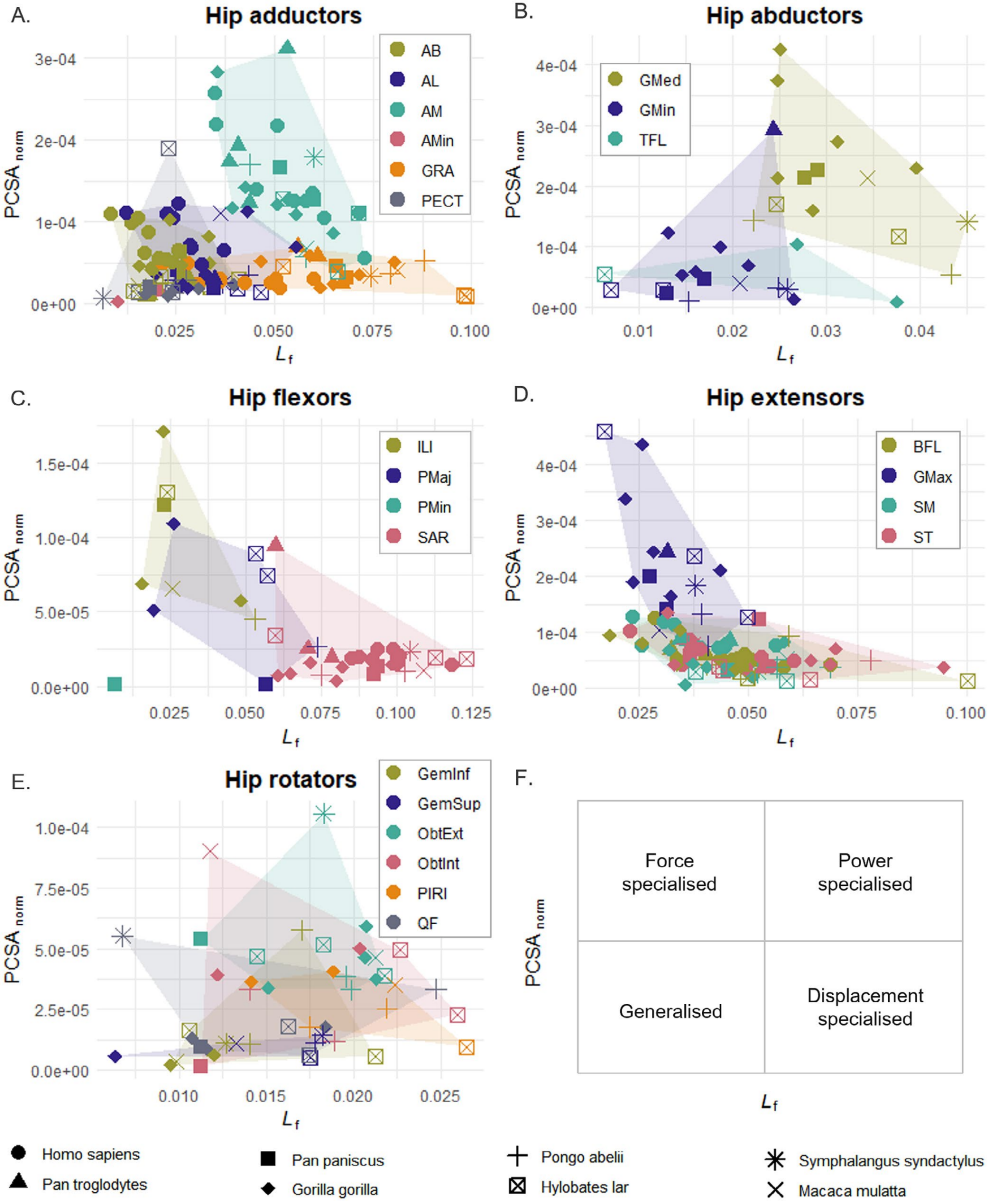
Functional space plots of the muscles that predominantly cross the hip, demonstrating the relationship between normalized $L_{f}$ and PCSA_norm_. Muscles are grouped according to primary function. Available muscle data from all specimens (Table 1) are included (*n* = 30).

**FIGURE 4:**
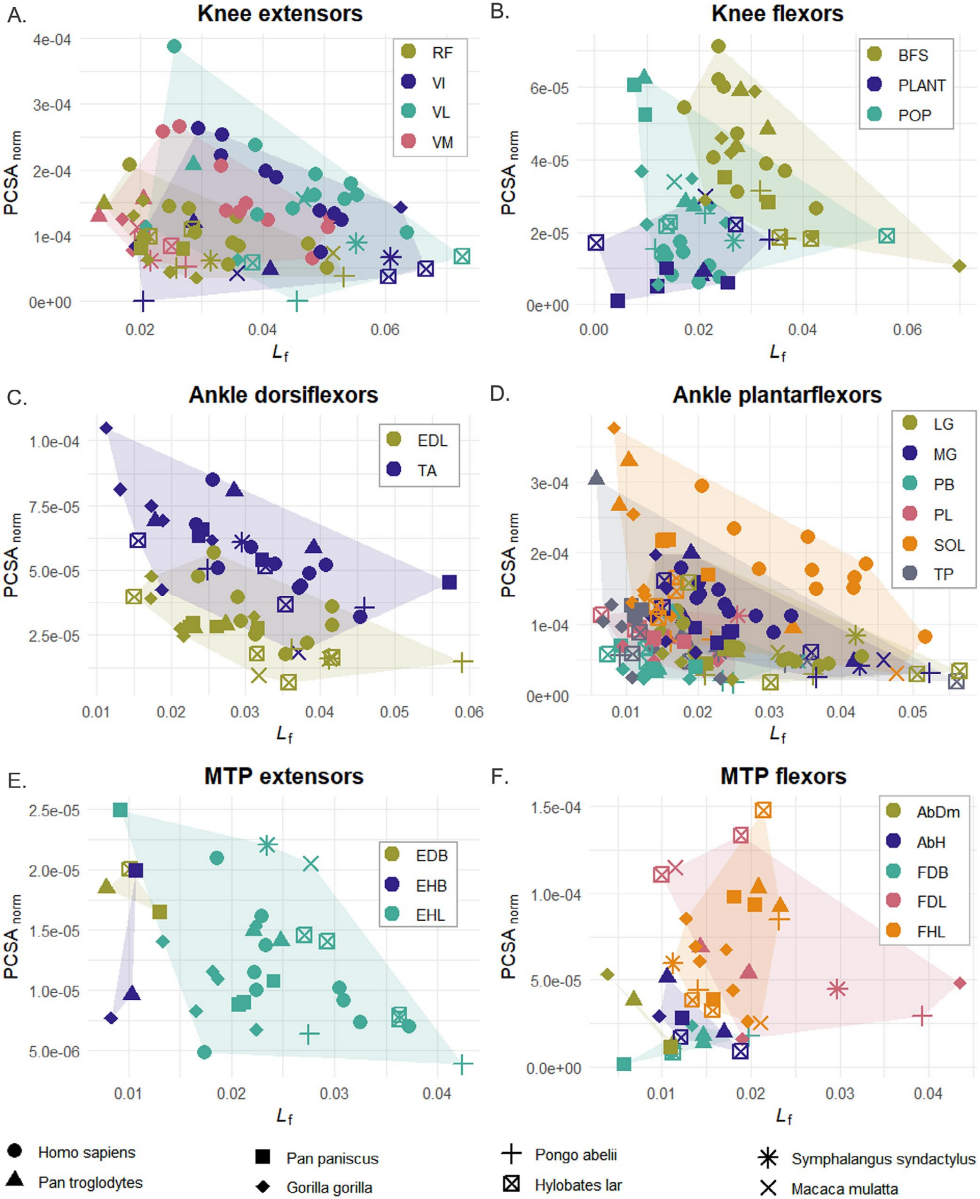
Functional space plots of the muscles that predominantly cross the knee, ankle and metatarsophalangeal joints, demonstrating the relationship between normalized $L_{f}$ and PCSA_norm_. Muscles are grouped according to primary function. Available muscle data from all specimens (Table 1) are included (*n* = 29).

We examined the relationship between $L_{f}$ and PCSA_norm_ to assess how muscles are functionally adapted (Figures 3 and 4). Muscles with a large PCSA_norm_ and short $L_{f}$ are typically specialized for force production, while those with both large PCSA_norm_ and long $L_{f}$ are suited for power generation. A small PCSA_norm_ combined with long $L_{f}$ suggests a bias toward specialization for range of motion (displacement), and muscles with both small PCSA_norm_ and short $L_{f}$ are less strongly differentiated along the force‐velocity axes and may function as stabilizers or generalists (Charles et al. 2020; Payne et al. 2006a; Charles et al. 2022; Wiseman et al. 2024). “Displacement‐oriented” is used interchangeably with velocity‐oriented architecture, as both reflect increased fiber length and associated capacity for greater muscle shortening velocity and excursion.

Across the dataset, most muscles showed broadly conserved specialization patterns. For example, the QF, PLANT, PB, and AB muscles exhibited relatively short $L_{f}$ and low PCSA_norm_ across all species. These muscles appeared functionally stable, with only minor interspecies variation in architectural scaling. Similarly, hip adductors (AM, AB, AL, GRA; Figure 3A) clustered tightly in functional space, indicating that these muscles have been evolutionarily conserved and maintain similar functional roles across primates. The AB and AL exhibited generalist architecture in all species (low $L_{f}$ and low PCSA_norm_). The GRA was displacement‐specialized in the gibbon, orangutan and chimpanzee, and acted more generally in the other species.

The hip abductors showed comparable organization: the GMin were generalists apart from the chimpanzee who was characterized as having a larger PCSA_norm_ (Figure 3B). The GMed tended toward displacement specialization in the gibbon, siamang, bonobo, and orangutan, but was instead power‐specialized in the gorilla.

In the hip flexors (Figure 3C), the SAR was displacement‐specialized in all taxa except chimpanzees, where it shifted slightly toward force production; PMaj was more generalist, and ILI was force‐specialized. Among hip extensors (Figure 3D), BFL, SM and ST were typically more displacement‐specialized, while GMax was generally intermediate with small PCSA_norm_ and short $L_{f}$ across most species, but was more force‐specialized (i.e., larger PCSA_norm_ with short $L_{f}$) in gorillas and gibbons.

Deep hip rotators were primarily displacement‐specialized in gibbons, gorillas, and orangutans with longer $L_{f}$ and smaller PCSA_norm_, but force‐specialized in siamangs with larger PCSA_norm_ and short $L_{f}$ (Figure 3E).

Among the knee extensors, there were few differences between species. Gibbons showed notably higher $L_{f}$ values for the VI and VL (Figure 4A), whilst chimpanzees were generally characterized by smaller $L_{f}$ values. Humans generally had slightly elevated PCSA_norm_ values across all knee extensors, with orangutans generally exhibiting smaller PCSA_norm_. No clear trends in species‐specific muscle function were evident in this muscle group.

The knee flexors displayed some interspecific variation (Figure 4B). BFS and POP were more force‐specialized in humans, bonobos and gorilla. In the other species, these muscles typically have smaller $L_{f}$ and smaller PCSA_norm_. The PLANT had small $L_{f}$ and PCSA_norm_ values across all species.

In the ankle dorsiflexors, the EDL exhibits longer $L_{f}$ with smaller PCSA_norm_ in orangutans versus the other species (Figure 4C). Gorillas have a force‐specialized TA (small $L_{f}$ and large PCSA_norm_), whilst the other species vary in the lengths of their TA with some species having a longer $L_{f}$ and others shorter $L_{f}.$

In the ankle plantarflexors, the PB, PL, and TP had small PCSA_norm_ and short $L_{f}$ across all species, whilst the LG, MG and SOL (triceps surae group) had elevated PCSA_norm_ (Figure 4D). There was large intra‐variability in the *Mm. triceps surae* of humans, but these human muscles did tend to have an elevated PCSA_norm_ versus the other species. Macaques, on the other hand, exhibited low PCSA_norm_ with long $L_{f}$ in the *Mm. triceps surae*, suited for velocity‐production. The gibbon and siamang had a similar configuration in only the LG and MG muscles, not the SOL.

Few observable differences between species were observed in the MTP extensors (Figure 4E). The EHL in orangutans was characterized by longer $L_{f}$ with small PCSA_norm_, whilst siamangs and macaques had the opposite pattern. Gorilla EHLs were generally intermediate, consistent with generalist function, while gibbon EHLs displayed traits indicative of displacement‐specialization.

In the FDL, gibbons, macaques and chimpanzees have a large PCSA_norm_ and short $L_{f}$, whilst the other species all have the opposite (Figure 4F). $L_{f}$ remains short in all species in the FHL, but PCSA_norm_ varies from small (macaques) to large (chimpanzees). All other muscles acting around the toes (AbDm, AbH, FDB) have both small PCSA_norm_ and short $L_{f}$.

When architectural distributions are visualized at the species level (Figure S1), several broad patterns emerge. African apes (including *Homo sapiens* ) show a consistent shift of ankle plantarflexors toward the high‐PCSA, short fascicle length region of morphospace, indicative of greater force‐oriented architecture, relative to other primates. In contrast, macaques tend to exhibit more generalized ankle plantarflexor configurations.

Across all taxa, hip flexors are positioned toward longer fascicle lengths and lower PCSA, consistent with a velocity‐oriented architectural profile. A distinct shift toward more force‐oriented knee extensor architecture is observed only in *Homo sapiens* , whereas other species retain more generalized or velocity‐biased configurations.

Macaques and siamangs do not exhibit clear clustering in the force‐dominated region for any functional group, instead occupying predominantly generalized or velocity‐oriented areas of morphospace. Finally, gorillas, bonobos, and gibbons display a comparatively broader spread of hip extensor architectures, indicating greater within‐group architectural diversity.

### 3.4 Differences in Relative Tendon Length Across the Hind Limb

**FIGURE 5:**
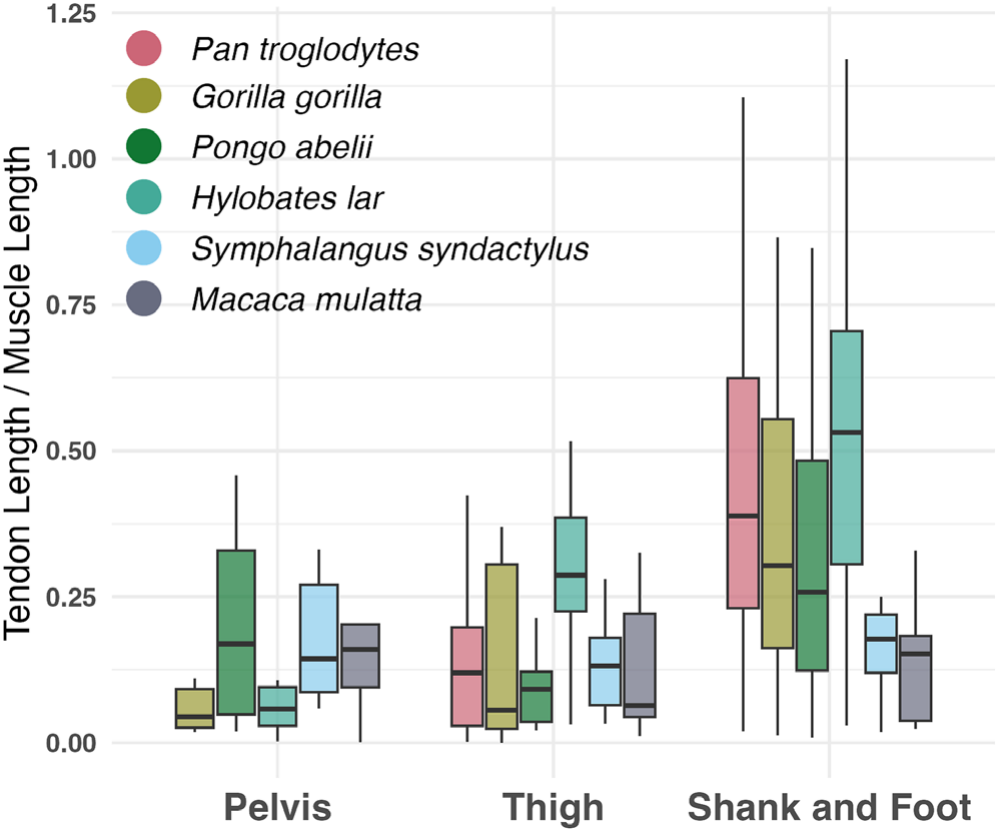
Relationship of tendon length to muscle length. Only specimens dissected in this study are included (*n* = 6) due to data availability.

Across all species, tendon length ratios increased distally, with the highest values observed in the ankle and foot muscles (Figure 5). Hip muscles consistently exhibited the lowest ratios, reflecting short tendons in proximal limb segments, whereas distal muscles are characterized by having relatively longer tendons. This distal increase was supported statistically, with a significant main effect of joint (two‐way ANOVA: df = 3, *F* = 5.89, *p* = 0.0007), and post hoc tests confirming that the foot had significantly higher tendon length ratios than the hip (*p* = 0.001), knee (*p* = 0.0015), and ankle (*p* = 0.0048).

Species differences were comparatively modest (species: df = 5, *F* = 2.34, *p* = 0.043), and no species × joint interaction was detected (*F* = 0.63, *p* = 0.85), indicating that species did not differ systematically at specific joints. Most pairwise contrasts were non‐significant after correction, with the only detected difference being slightly lower tendon length ratios in macaques compared with gibbons (Δ = −0.39, *p* = 0.030). Accordingly, visually elevated ankle ratios (Figure 5) in gibbons and siamangs were not found to be statistically different after correcting for multiple comparisons. The overall species effect was weak (ANOVA: *F* = 2.34, *p* = 0.043), and only one pairwise contrast (macaques < gibbons) reached significance. Overall, these results indicate that tendon length variation is driven primarily by joint‐level (proximal–distal) differences rather than strong species‐specific specializations.

## 4 Discussion

### 4.1 Functional Adaptation of Muscle Architecture

Comparative analyses of muscle morphology across the hind limb reveal a clear mosaic of functional adaptations. Generally, muscle origins and insertions are broadly conserved across species, which likely reflects strong biomechanical constraints on joint function, as the geometry of the major hip extensors, abductors, and rotators underpins effective joint stabilization across diverse locomotor regimes (Aerts et al. 2018; Hogervorst and Vereecke 2015; Payne et al. 2006b; Vereecke et al. 2005). However, the variability observed in the deep hip rotators and adductor complex, including the inconsistent presence of *Mm. obturator*, *gemelli*, and AMin, suggests that these smaller stabilizers are developmentally and functionally less constrained, likely adapting to subtle shifts in habitual posture and limb loading. Proximal muscles associated with hip stabilization and force transmission were largely conserved in their architectural profiles, whereas distal muscles showed greater interspecific variability in both muscle and tendon composition. This distal flexibility was most evident in gibbons, which exhibited high tendon ratios and velocity‐biased muscle architectures, consistent with enhanced elastic and displacement‐oriented function necessary for a highly arboreal lifestyle (Channon et al. 2009; Granatosky 2018).

Importantly, our findings underscore the importance of incorporating pennation angle into biomechanical analyses of primate musculature, especially in comparative contexts, in contrast to a few studies that have suggested pennation angle may be redundant in force‐production estimates (Lieber 2022; Martin et al. 2020). While some models have assumed that fascicle length and PCSA alone sufficiently capture a muscle's mechanical potential (Leischner et al. 2018), our comparative data reveal that pennation angle can substantially alter architectural interpretation, particularly in species with high pennation and short $L_{f}$, such as chimpanzees who exhibit consistently large pennation angles across both thigh and shank muscles, coupled with short $L_{f}$. This configuration suggests a mechanical bias toward maximizing force output through increased fiber packing, at the expense of contraction velocity or range (Zajac 1989). High pennation may serve as a compensatory mechanism for supporting large body mass and generating powerful hind limb extension during climbing and/or terrestrial locomotion. Notably, these architectural traits are not mirrored in the large‐bodied gorillas or orangutans which show lower pennation and longer $L_{f}$, thus reflecting divergent mechanical strategies aligned with increased reliance on other locomotory behaviors, such as, for example, suspensory locomotion in the orangutan (e.g., Granatosky 2018). While many muscles show conserved configurations, others diverge markedly in $L_{f}$ and PCSA_norm_ reflecting functional specialization. Muscles involved in postural support and load‐bearing (e.g., GMax, PECT, VM, GRA) consistently exhibited small $L_{f}$ and larger PCSA_norm_ across species, suggesting evolutionary stability in force‐specialized roles. In contrast, SAR exhibited a tendency toward displacement‐speciality, indicating adaptation to large range of motion in all species.

One of the most striking findings in this study is the presence of architectural similarities in different taxa, such as similar properties in ankle musculature. Whilst there were a few species that were more suited for force or displacement activities in select muscles (i.e., more force‐producing *triceps surae* in gorillas), generally most species overlapped in muscle function. Despite their divergent phylogenetic positions and locomotor ecologies (i.e., Granatosky 2018), these species share common functional demands (Aerts et al. 2018; Channon et al. 2009; Manduell et al. 2011; Payne et al. 2006b; Thorpe and Crompton 2005, 2006; Thorpe et al. 2007; Vereecke et al. 2006, 2005), such as the need for controlled limb support, balance, and versatile movement across different substrates, which likely contributed to convergent muscle specialization. This convergence supports the hypothesis that locomotor ecology exerts a stronger influence on muscle architecture than phylogenetic relatedness alone (Marchi et al. 2018), although phylogeny and function covary in these lineages, making their relative contributions difficult to discern. Marchi et al. (2018) found that hind limb muscle configuration in primates aligns more closely with locomotor demands, such as leaping and climbing, than with evolutionary lineage. However, because phylogeny and function covary in these lineages, their relative contributions cannot be fully separated.

Such convergence has important implications for reconstructing the soft tissues of fossil hominoids and understanding their functional capacities from an evolutionary perspective. For example, the frequent use of human and chimpanzee muscle data as proxies for ancestral states in the reconstruction of hominin locomotion (Bates et al. 2025; O'Neill et al. 2024; Wiseman 2023; Wiseman et al. 2024) has provided valuable biomechanical baselines, especially given their well‐characterized anatomy and locomotor repertoires. However, our results suggest that these models may not fully capture the diversity of ancestral muscle function, particularly if architectural traits are more plastic and ecologically driven than previously assumed. For example, we propose that the smaller PCSA_norm_ in the knee extensors in chimpanzees relative to humans may reflect differences in locomotor ecology, with humans showing adaptations for sustained terrestrial bipedalism and chimpanzees retaining traits supportive of greater arboreal behaviors (i.e., Crompton et al. 2008, 2010). However, the architectural configurations observed in humans and chimpanzees do not necessarily reflect the ancestral condition of early bipeds, who may have instead had combined intermediate features to accommodate a mix of terrestrial and arboreal locomotor behaviors, consistent with interpretations of their skeletal function. This highlights the importance of considering both derived adaptations and functional convergence when interpreting muscle architecture in an evolutionary context.

Our findings advocate for a more ecologically contextualized comparative framework for reconstructing soft tissues in fossil specimens. We propose that muscle architecture should not be constrained to just one or two analogous ‘brackets’ (Demuth et al. 2023; Molnar and Diogo 2021; Wiseman et al. 2024; Witmer 1995), but should also consider potential ecological analogues. Our results highlight that locomotor ecology, rather than phylogenetic relatedness alone, strongly influences muscle architecture. In the case of muscle reconstructions in fossil hominins, reliance on a limited phylogenetic bracket may obscure ecologically structured variation; instead, a broader and more ecologically representative comparative sample is required. Expanding comparative datasets to include additional locomotor strategies and taxa could further test and refine this ecological signal.

### 4.2 Limitations

Not all muscles were dissected in every specimen for a variety of aforementioned reasons, limiting the completeness of some functional group analyses. Nevertheless, our results support the view that muscle properties vary substantially within and between groups.

Muscle architecture is influenced by a range of individual factors, including age, activity level, and pathology (Létocart et al. 2021). The specimens included in this study provide a unique opportunity to explore variation in muscle architecture across life histories and loading regimes. By combining these novel data with published measurements from the literature, we begin to build a more comprehensive picture of primate muscle architecture. Moving forward, openly sharing muscle data using consistent collection protocols will allow different research groups to compile larger comparative datasets, enhancing our understanding of how age, sex, and ecological factors shape musculoskeletal variation.

Recent work has shown that hominoids may exhibit different scaling relationships between body mass and muscle cross‐sectional area (Warrener 2024), likely reflecting differences in habitual loading regimes associated with locomotor behavior. As all extant hominoids are locomotor specialists to varying degrees, some interspecific differences observed here may reflect lineage‐specific scaling trajectories in addition to functional adaptation. Although architectural variables were normalized to facilitate comparison, the limited sample sizes available for rare primate cadavers prevented explicit modeling of lineage‐specific allometric relationships. Future studies with larger within‐species samples will be necessary to disentangle the relative contributions of body size, locomotor ecology, and phylogenetic history.

## 5 Conclusion

Our study highlights the importance of a broader comparative framework for understanding primate locomotor diversity. By presenting new detailed dissection data from six primate species and integrating comparative results across multiple studies (Charles et al. 2019; Myatt et al. 2012; Oishi et al. 2009; Payne et al. 2006a; Vereecke et al. 2005), we demonstrate that locomotor ecology plays a key role in shaping hind limb muscle architecture, both among closely and distantly related taxa. Our findings suggest that ecological factors, such as substrate use and locomotor repertoire, likely exert a strong influence on muscular design.

## Author Contributions

**Emma Guimaraes:** investigation, writing – original draft, writing – review and editing, visualization, methodology, formal analysis. **Evie Vereecke:** investigation, writing – original draft, writing – review and editing, resources, data curation, methodology. **Ashleigh L. Wiseman:** conceptualization, investigation, funding acquisition, writing – original draft, writing – review and editing, visualization, methodology, supervision.

## Funding

This work was supported by the AABA Cobb Professional Development Grant, a Leverhulme Trust Early Career Fellowship (grant no. ECF‐2021‐054), and the Isaac Newton Trust (Project_21.08(a)) at the University of Cambridge, and further supported by the STEPS project (ERC, Grant No. 101219207), awarded to A.L.W.

## Ethics Statement

Ethical approval for primate dissections was provided by the Animal Ethics Committee of the KU Leuven, Belgium (approval codes M005/2023, M006/2023), and all procedures complied with EU regulations.

## Conflicts of Interest

The authors declare no conflicts of interest.

## Supporting information


**Figure S1:** Functional space plots of all pelvic and hind limb muscles within each species, illustrating the relationship between $L_{f}$ and PCSA_norm_. Muscles are grouped according to primary function (Table 3). Muscle data from all specimens (Table 1) are included.

**Data S1:** ajpa70329‐sup‐0002‐DataS1.xlsx.

**Data S2:** ajpa70329‐sup‐0003‐DataS2.docx.

## Data Availability

Measured dissection parameters are provided in the Supporting Information.
