# Supplementary figures and images for: Functional Differences in Muscle Architecture Across the Pelvis and Hind Limb of Primates
Source: Am J Biol Anthropol. 2026 Jul 31;190(4):e70329. doi: 10.1002/ajpa.70329 (PMC13425262; doi:10.1002/ajpa.70329)

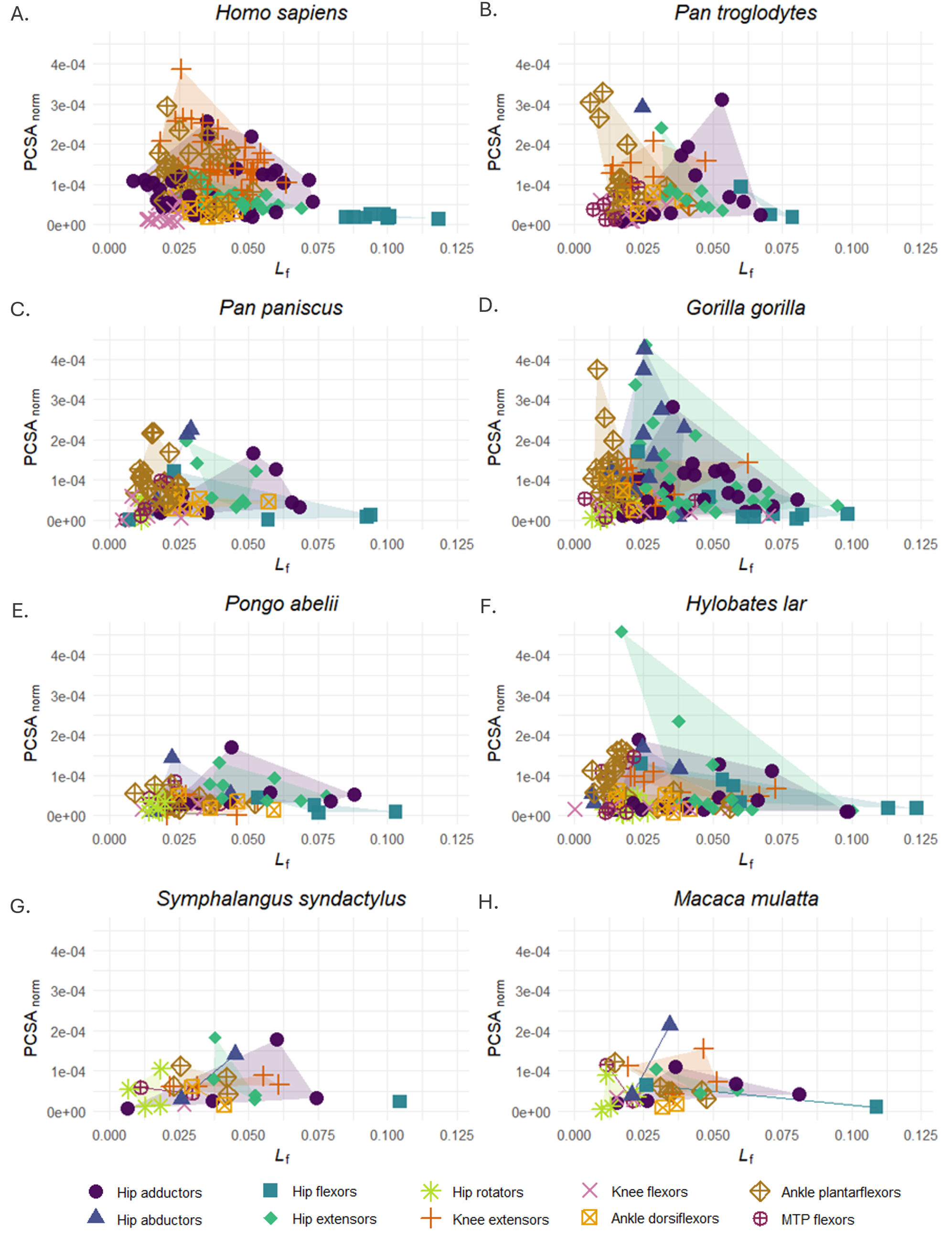

Supplement: Supplementary file 1 — Figure S1: Functional space plots of all pelvic and hind limb muscles within each species, illustrating the relationship between Lf and PCSAnorm. Muscles are grouped according to primary function (Table 3). Muscle data from all specimens (Table 1) are included. [file AJPA-190-e70329-s003.tif]
