# Supplementary material for: Functional Differences in Muscle Architecture Across the Pelvis and Hind Limb of Primates
Source: Am J Biol Anthropol. 2026 Jul 31;190(4):e70329. doi: 10.1002/ajpa.70329 (PMC13425262; doi:10.1002/ajpa.70329)
Supplement: Supplementary file 3 — Data S2: ajpa70329‐sup‐0003‐DataS2.docx. [file AJPA-190-e70329-s002.docx]

**Functional differences in muscle architecture across the pelvis and lower limb of primates**

**Supplementary Information 2**

Emma Guimaeres^1^, Evie Vereecke^2^, Ashleigh L Wiseman^1,3^

^1^ Department of Anthropology, University College London, London, UK.

^2^ Department of Development and Regeneration, KU Leuven Campus Kulak, Kortrijk, Belgium.

^3^ McDonald Institute for Archaeological Research, University of Cambridge, Cambridge, UK.

**1. Muscle homologies**

Here, we report differences in muscle attachment sites. Refer to Section 2 for differences in architecture. Further see Wiseman et al. 2026 for further discussions on muscle homologies in these taxa.

*Scansorius*

This muscle was present in the macaque and siamang only, originating from the anterior superior iliac spine (ASIS) and inserting on the greater trochanter. The pattern suggests a retained primitive configuration in cercopithecoids and some lesser apes, with loss in taxa where the hip flexor complex is reorganised.

*Obturator internus (ObtInt)*

Present in all taxa, excluding the siamang. Macaques possess a well‑developed *obturator internus* arising from the obturator foramen, including an additional slip. The muscle in the remaining species originates from the obturator foramen and inserts into the trochanteric fossa.

*Obturator externus (ObtExt)*

This muscle was present in all taxa, arising from the medial obturator foramen and inserting into the trochanteric fossa.

*Adductor magnus (AM)*

Across all taxa, adductor magnus originates from the inferior pubic ramus and ischial tuberosity. Insertions vary: macaques, orangutans, gorillas, chimpanzees and humans insert on the linea aspera and adductor tubercle, whereas in the gorilla is additionally extended to the tibial epicondyle.

*Adductor longus (AL)*

Origins are conserved across species, from the anterior superior pubic ramus. Insertions consistently lie along the middle third of the linea aspera.

*Adductor brevis (AB)*

This muscle shows substantial variation. Macaques and siamangs both possess two heads from the superior pubic ramus, inserting on the linea aspera. Gibbons lack the muscle entirely. Orangutans, chimpanzees, and humans retain a single origin from the body/inferior ramus of the pubis with insertion on the linea aspera.

*Adductor minimus (Amin)*

Absent in the macaque, gibbon, siamang, orangutan, and human. Present in the chimpanzee and gorilla, arising from the superior/inferior pubic ramus and inserting on the dorsal femoral shaft.

*Psoas major (PM)*

Conserved across taxa, arising from T12–L5 vertebrae and inserting on the lesser trochanter.

*Pectineus (PECT)*

The macaque, gibbon, gorilla, chimpanzee, and human showed the typical origin from the superior pubic ramus and insertion on the pectineal line of the femur. The orangutan had a fused pectineus with adductor longus.

*Piriformis (PIRI)*

The gibbon, orangutan, gorilla, chimpanzee, and human showed the typical origin on the anterior sacrum and insertion on the greater trochanter. Siamangs exhibit fusion of piriformis with gluteus medius.

*Quadratus femoris (QF)*

Absent in the macaque. Present in all other species, arising from the ischial tuberosity and inserting on the intertrochanteric crest.

*Gemellus superior (GemSup)*

Absent in the gorilla. Present in all other species, arising from the ischial spine and inserting into the trochanteric fossa.

*Gemellus inferior (GemInf)*

Present in all species. Arises from ischial spine in the macaque, but from the ischial tuberosity in all other species and inserting into the trochanteric fossa.

*Gluteus maximus (GMax)*

Absent in macaques. Present in all other species, with origins from the ilium, sacrum, coccyx, and sacrotuberous ligament. Insertions include the iliotibial tract and posterolateral femur. Orangutans and gorillas show expanded origins including the posterior iliac crest and ischial tuberosity.

*Gluteus superficialis*

Present in macaques, arising from the iliac crest and sacrum and inserting on the gluteal tuberosity. Assumed homologous with the GMax.

*Tensor fasciae latae (TFL)*

Arising from the ASIS and inserting into the iliotibial tract. In the siamang, the proximal region is fused with gluteus maximus.

*Gluteus medius (GMed)*

Conserved across all taxa, arising from the lateral ilium and inserting on the lateral surface of the greater trochanter. Siamangs show partial fusion with piriformis.

*Gluteus minimus (GMin)*

Present in all taxa, arising from the dorsolateral ilium and inserting on the anterior surface of the greater trochanter.

*Ischiofemoralis (IF)*

Absent in the macaque, gibbon, siamang, gorilla and human. Present in chimpanzee and orangutan, with origins near the adductor magnus insertion (ischial tuberosity) and insertions on the distal femoral shaft, semi-fused with vastus lateralis in the orangutan or gluteus maximus in the chimpanzee.

*Iliacus (ILI)*

Conserved across taxa, arising from the iliac fossa and inserting on the lesser trochanter.

*Sartorius (SAR)*

Arises from the ASIS and inserts on the medial tibial shaft across all taxa, superficial to gracilis.

*Popliteus (POP)*

Consistently arises from the lateral femoral condyle and inserts on the posterior tibia above the soleal line across all species.

*Rectus femoris (RF)*

Originates from the AIIS and inserts via the patellar tendon in all taxa.

*Vastus lateralis (VL), intermedius (VI), medialis (VM)*

All three vasti show conserved origins on the femoral shaft (greater trochanter/linea aspera for lateralis; ventral shaft for intermedius; medial shaft for medialis) and insert via the patellar tendon.

*Gastrocnemius (medial and lateral heads) (MG and LG)*

Both heads originate from their respective femoral condyles and insert on the posterior calcaneus via the Achilles tendon.

*Biceps femoris (long head) (BFL)*

Originates from the ischial tuberosity in all species and inserts in to the tibial head, tibial tuberosity, and fibula.

*Biceps femoris (short head) (BFS)*

Arises from the dorsolateral femoral shaft and inserts on the fibular head in most taxa. The chimpanzee had an additional insertion into the tibial shaft.

*Gracilis (GRA)*

Originates from the body and inferior pubic ramus and inserts on the ventromedial tibia in all species.

*Semimembranosus (SM)*

Originates from the ischial tuberosity in all specimens. Insertions vary: macaques show an accessory head inserting proximally on the femoral condyle, whereas all other specimens insert medially on the tibial tuberosity or tibial condyle.

*Semitendinosus (ST)*

Arises from the ischial tuberosity and inserts on the medial proximal tibia, distal to gracilis.

*Flexor digitorum longus (FDL)*

Arises from the posterior tibial shaft in all taxa. Insertions vary: macaques and humans insert on distal phalanges II–V; the chimpanzee showed fusion with flexor hallucis longus, altering digital distribution.

*Flexor digitorum brevis (FDB)*

Arises from the medial process of the calcaneus in all species. Insertions vary: in the human and the orangutan, it inserted on middle phalanges II–V. In the gorilla, chimpanzee, macaque, siamang and gibbon, it instead inserted into the interosseous membrane and dorsal fibula.

*Flexor hallucis longus (FHL)*

Consistently originates from the interosseous membrane and dorsal surface of the fibula across all taxa. In the macaque, siamang and gibbon, it inserts on the base of the hallux and also contributes tendons to digits II–IV via fusion with flexor digitorum longus. In the human, gorilla and orangutan, it has a single insertion on the distal phalanx of the hallux. The chimpanzee exhibits dual insertions: on digit 1 and digit 2, with fusion to flexor digitorum longus.

*Flexor hallucis brevis (FHB)*

Origins vary across midfoot structures: cuneonavicular ligament (macaque, gibbon, siamang), intermediate cuneiform (orangutan, gorilla, chimpanzee), and the cuboid and lateral cuneiform (human). Inserts on the proximal phalanx of the hallux in all taxa.

*Soleus (SOL)*

Arises from the fibular head and shaft and inserts on the posterior calcaneus via the Achilles tendon.

*Tibialis posterior (TP)*

Arises from the interosseous membrane and adjacent tibia/fibula. Insertions vary slightly across midfoot bones but consistently include navicular and cuneiforms.

*Plantaris (PLANT)*

Absent in the macaque, orangutan, gorilla, and human. Present in siamang, gibbon, and chimpanzee, arising from the lateral femoral supracondylar line and inserting on the posterior calcaneus.

*Extensor digitorum longus (EDL)*

Arises from the lateral tibial condyle and fibula, inserting on digits II–IV.

*Fibularis (peroneus) longus (PL)*

Arises from the upper lateral fibula and inserts on the base of the first metatarsal and medial cuneiform (humans only).

*Tibialis anterior (TA)*

Arises from the lateral tibia. Insertions vary slightly: macaques and humans insert on the medial cuneiform and first metatarsal at proximal end.

*Extensor hallucis longus (EHL)*

Arises from the medial fibula and interosseous membrane, inserting on the distal phalanx of the hallux.

*Extensor hallucis brevis (EHB)*

Arises from the dorsal calcaneus. Insertions vary: chimpanzee had slips to digits 1–4.In all other taxa, there was a single insertion to the base of proximal phalanx of the hallux.

*Fibularis (peroneus) brevis (PB)*

Arises from the distal lateral fibula and inserts on the tuberosity of the fifth metatarsal.

*Abductor digiti minimi (ADM)*

Arises from the calcaneal tuberosity and inserts on the base of the proximal phalanx of digit 5.

**2. Results: inter-specific variation in muscle architecture.**

To assess whether the architectural trends observed in the main dataset were upheld across a broader sample, we incorporated additional published data from *Gorilla gorilla*, *Hylobates lar*, *Pongo abelii*, *Homo sapiens* and *Pan paniscus* (Charles et al., 2020; Myatt et al., 2012; Oishi et al., 2009; Payne et al., 2006). Inclusion of additional dissection material allows us to explore inter-specific variation. Overall, the expanded dataset preserved the general organisation of muscle specialisation described in the main text. Hip adductors (AM, AB, AL, PECT) remained tightly clustered, with AM consistently power-specialised and AB/AL showing generalist architecture. The PECT retained its force-specialised profile in *Hylobates lar*. Similarly, SAR, SM, ST, and GMax maintained their displacement- or intermediate specialisations, with only minor shifts in Lf:PCSA ratios across added specimens. Deep rotators continued to show displacement bias in most taxa, with force-specialisation restricted to *Symphalangus syndactylus*.

The distal muscles tended to be more inter-specific variable. SOL, MG and LG muscles (the *M. triceps surae*) was quite variable within species, although there was a tendency for the *Macaca mulatta* to have a generalised specialisation of this muscle group, whilst *Homo sapiens* exhibited a mix of force- and velocity- specialisation. We hypothesise that these architectural measurements to do not take into consideration the sedentary versus active lifestyle of modern populations.

Other muscles retained broad profiles. For example, the TA and EDL retained displacement-biased profiles. The FHL and FDL muscles showed consistent generalist or displacement-specialised architecture, with minimal interspecies divergence. Among knee extensors, RF and VL preserved their divergent scaling patterns: RF remained intermediate or force-specialised in most taxa, while VL was velocity-specialised in *Pongo abelii* but force-specialised elsewhere. Knee flexors (e.g., BFL, BFS, GRA) showed greater architectural dispersion, particularly in *Hylobates lar*, where elevated Lf:PCSA ratios suggest enhanced velocity capacity.

Overall, the inclusion of additional specimens increased trait-space overlap and introduced greater architectural spread, particularly in muscles with previously narrow ranges. These shifts likely reflect both biological variation and methodological heterogeneity across studies.

To assess whether species differed in the degree of architectural variation among sampled muscles, we performed a multivariate dispersion analysis using the *betadisper*() function in R. This approach calculates the distance of each observation to its species centroid in trait space, based on Euclidean distances derived from Lf and PCSA values. We tested for differences in dispersion using ANOVA and permutation-based pairwise comparisons (999 permutations).

The ANOVA revealed no significant differences in dispersion across species (F₁₁,₇₄₈ = 1.68, p = 0.074), indicating that the overall spread of muscle architecture within species was broadly comparable. Permutation tests supported this finding (p = 0.112), with most pairwise comparisons yielding non-significant results. However, *Homo sapiens* exhibited significantly greater dispersion than *Gorilla gorilla* (p = 0.009). No other species pairs showed consistent differences in trait-space spread.

These results suggest that the architectural trends described in the main text are not driven by unequal within-species variation. We conclude that the core functional patterns remain robust across taxa.

Additional References:

Charles, J. P., Grant, B., D'Aout, K., & Bates, K. T. (2020). Subject-specific muscle properties from diffusion tensor imaging significantly improve the accuracy of musculoskeletal models. *J Anat*, *237*(5), 941-959. <https://doi.org/10.1111/joa.13261>

Myatt, J. P., Crompton, R. H., Payne-Davis, R. C., Vereecke, E. E., Isler, K., Savage, R., D’Août, K., Günther, M. M., & Thorpe, S. K. S. (2012). Functional adaptations in the forelimb muscles of non-human great apes. *Journal of Anatomy*, *220*(1), 13-28. <https://doi.org/https://doi.org/10.1111/j.1469-7580.2011.01443.x>

Oishi, M., Ogihara, N., Endo, H., Komiya, T., Kawada, S.-i., Tomiyama, T., Sugiura, Y., Ichihara, N., & Asari, M. (2009). Dimensions of the Foot Muscles in the Lowland Gorilla. *Journal of Veterinary Medical Science*, *71*(6), 821-824. <https://doi.org/10.1292/jvms.71.821>

Payne, R. C., Crompton, R. H., Isler, K., Savage, R., Vereecke, E. E., Günther, M. M., Thorpe, S. K. S., & D’Août, K. (2006). Morphological analysis of the hindlimb in apes and humans. I. Muscle architecture. *Journal of Anatomy*, *208*(6), 709-724. <https://doi.org/10.1111/j.1469-7580.2005.00433.x-i1>

Wiseman, A. L., van Beesel, J. B., & Vereecke, E. (2026). Comparative analysis of primate hind limb muscle moment arms using subject-specific three-dimensional musculoskeletal models. *Royal Society Open Science*.<https://doi.org/10.1098/rsos.260107>
